# Genetic diversity and novel lineages in the cosmopolitan copepod *Pleuromamma abdominalis* in the Southeast Pacific

**DOI:** 10.1038/s41598-019-56935-5

**Published:** 2020-01-24

**Authors:** Carolina E. González, Erica Goetze, Rubén Escribano, Osvaldo Ulloa, Pedro Victoriano

**Affiliations:** 1 Graduate Program in Oceanography, Department of Oceanography, University of Concepción, PO Box 160, Barrio Universitario s/n Concepción, Concepción, 4030000 Chile; 2 Instituto Milenio de Oceanografía (IMO) and Department of Oceanography, Faculty of Natural and Oceanographic Sciences, Universidad de Concepción, PO Box 160 C, Barrio Universitario s/n Concepción, Concepción, 4030000 Chile; 3 Department of Oceanography, University of Hawaii at Manoa, 1000 Pope Road, Honolulu, Hawaii USA; 4 Department of Zoology, Faculty of Natural and Oceanographic Sciences, University of Concepción, PO Box 160 C, Barrio Universitario s/n Concepción, Concepción, 4030000 Chile

**Keywords:** Biodiversity, Marine biology

## Abstract

Across boundary currents, zooplankton are subject to strong oceanographic gradients and hence strong selective pressures. How such gradients interact with the speciation process of pelagic organisms is still poorly understood in the open ocean realm. Here we report on genetic diversity within the pelagic copepod *Pleuromamma abdominalis* in the poorly known Southeast Pacific region, with samples spanning an ocean gradient from coastal upwelling to the oligotrophic South Pacific Subtropical Gyre. We assessed variation in fragments of the mitochondrial (mt) genes cytochrome *c* oxidase subunit I (COI) and Cytochrome *b* as well as in the nuclear internal transcribed spacer (ITS) region and 28 S rRNA. Phylogenetic analyses revealed the presence of 8 divergent lineages occurring across the gradient with genetic distances in the range of 0.036 and 0.44 (mt genes), and GMYC species delimitation methods support their inference as distinct (undescribed) species. Genetic lineages occurring across the zonal gradient showed strong genetic structuring, with the presence of at least two new lineages within the coastal upwelling zone, revealing an unexpectedly high level of endemism within the Humboldt Current System. Multivariate analyses found strong correlation between genetic variation and surface chlorophyll-*a* and salinity, suggesting an important role for hydrographic gradients in maintaining genetic diversity. However, the presence of cryptic lineages within the upwelling zone cannot be easily accounted for by environmental heterogeneity and poses challenging questions for understanding the speciation process for oceanic zooplankton.

## Introduction

One of the largest and least explored regions of the open ocean is the Southeast Pacific (SEP). This region is globally important due to its role in controlling regional climate, its atmospheric and oceanic teleconnections at a global scale^1,2^, and due to the presence of one of the strongest and shallowest oxygen minimum zones in the global ocean^3,4^. This region exhibits high heterogeneity in hydrographic conditions, primarily along a zonal gradient from the coastal zone to the open ocean, with increasing mixed layer depth (40–150 m), sea surface temperature (14–20 °C), sea surface salinity (34.8–35.8) and surface dissolved oxygen concentration (1.5–4.5 mlL^−1^)^5,6^. This high zonal variability is also clearly manifested in productivity of surface waters from the highly productive coastal upwelling zone (Humboldt Current), across to the large ultra-oligotrophic South Pacific Subtropical Gyre (SPSG)^7–10^. Moreover, within this zonal gradient there is a changing predominance of different water masses promoting even greater hydrographic variability in this vast ecosystem. The SPSG is the largest subtropical gyre, with an areal extent of 18 × 10^6^ km^2,11^, and it has the lowest reported primary production rates (1–2 μg C L^−1^ day^−1^)^12^. The ultra-oligotrophic condition of the eastern SPSG contrasts with the very high productivity of the SEP upwelling system off the coast of Chile, Peru and Ecuador, also known as the Humboldt Current System (HCS), which is one of the most productive eastern boundary current ecosystems in the world^8,13^. Primary production in the upwelling zone reaches up to 19.9 g C m^−1^ d^−1^ ^14^. Upwelling-driven production extends zonally into a mesotrophic region, also known as the coastal transition zone^15^, which constitutes an intermediate region between the upwelling system and the oligotrophic region of the SPSG.

Oceanographic variability across the SEP zonal gradient is strongly correlated with changes in zooplankton abundance, biomass and distribution. Zooplankton biomass and abundance can decline by up to 3 orders of magnitude from the coastal zone to the SPSG^16^. Within the central gyre, small organisms (<500 μm),which numerically represent up to 65% of the zooplankton, are dominated by copepods, whereas other size fractions composed of chaetognaths, euphausiids and siphonophores make up a considerable part of the community^11,16^. In the coastal zone in contrast, larger-sized copepods often dominate numerical abundance^17^. In specific groups of zooplankton, for example siphonophores, tintinnids, and euphausiids, there is an increase in diversity and species richness towards the center of the gyre^6^. There also are endemic species within the Humboldt Current that are replaced by different species in the oceanic zone^18–20^. For example, the endemic copepod *Calanus chilensis* of the Humboldt Current^21^ appears to be replaced by the more widely distributed *Calanus australis* towards open ocean waters^19^. A similar pattern seems to occur with the endemic Humboldt Current *Euphausia mucronata*, which may be replaced by other euphausiids in the oligotrophic zone^20^. How the apparent environmental barriers across the zonal gradient, from the Humboldt Current to the SPSG, have influenced species distributions, population structure and genetic diversity within zooplankton species are poorly known for this ocean basin.

Planktonic copepods are the most species-rich taxonomic group of the mesozooplankton, with a large number of cryptic species reported^22–25^. Strong genetic structure among populations within plankton species also has been shown to occur from coastal to oceanic environments^26^. For example, in the cosmopolitan copepods *Eucalanus hyalinus*, *E. spinifer*, *Pleuromamma xiphias* and *Haloptilus longicornis*^27–29^ ocean regions with strong hydrographic gradients can act as ecological barriers to dispersal, causing strong genetic breaks among populations in distinct subtropical gyres and/or among ocean basins. Because biotic and abiotic characteristics of regions with strong zonal gradients may affect the development or survivorship of populations^22^, they can act as barriers to dispersal. Responses to these ocean gradients can be species-specific (idiosyncratic responses), due to the variable ecological requirements of each species^26^. Given that genetic divergence is commonly decoupled from morphological divergence in copepods^30,31^, and that broadly-distributed species may include lineages with different evolutionary trajectories^25^, detection of cryptic diversity in this group is fairly common.

Within the SEP, there are few copepod species that show broad distribution and high abundance across the zonal gradient. As a result, it is difficult to identify appropriate target species for studies aiming to elucidate the effect of hydrographic variability on genetic structuring within species. One of the few species that meets these requirements is *Pleuromamma abdominalis*, which has been described to inhabit preferably oceanic environments, has a cosmopolitan distribution in subtropical and tropical waters worldwide, and occurs in high abundance across the mesopelagic and epipelagic zones with active diel migratory behavior^32–37^. Within the SEP, seven species of *Pleuromamma* co-occur, including *P. borealis*, *P. gracilis*, *P. piseki*, *P. quadrungulata*, *P. robusta* and *P. xiphias*^35^. *P. abdominalis* can be clearly distinguished from other co-distributed species by the shape of the rostrum, the presence and number of denticles on proximal segments of the A1, body size, and in adult females by the 5^th^ swimming leg segmentation^38^. In recent studies conducted on this species, large intraspecific variation was reported in both morphological and genetic characters^39–41^. Prior work has reported the presence of endemic clades in certain oceanographic provinces, including subtropical waters of the South Pacific^41^.

**Figure 1:**
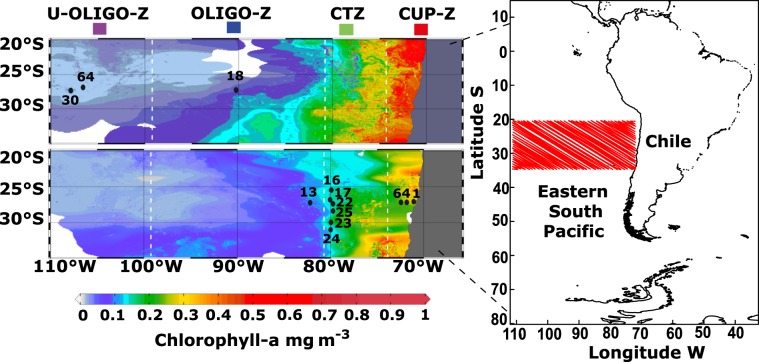
The Southeast Pacific where the CIMAR21 and CIMAR22 cruises were carried out in the years 2015–2016. Sampling stations with numbering as shown. The four oceanographic zones were defined by surface chlorophyll-a values: coastal upwelling zone (CUP-Z), the mesotrophic coastal transition zone (CTZ), the oligotrophic zone (OLIGO-Z) and the ultra-oligotrophic zone (U-OLIGO-Z). Colors shown for each zone are included in other figures to indicate these ocean regions.

In this work, we conducted a molecular study of the evolutionary lineages present within the nominal species *P. abdominalis* in the Southeast Pacific. Our objectives were to (1) identify the evolutionary lineages present in the SEP region, (2) evaluate whether they may be distinct, currently undescribed species, using multiple independently inherited markers, and (3) determine the potential effects of hydrographic variability across the SEP region on the distribution of these distinct evolutionary lineages. For this, we used samples collected across a zonal transect of the SEP (13 sites; Fig. 1, Table 1), and study genetic diversity within *P. abdominalis* using sequences from the mitochondrial genes cytochrome *c* oxidase subunit I (COI) and cytochrome *b* (CytB), as well as nuclear 28 S rRNA and the internal transcribed spacer (ITS) region.

**Table 1:** Sampling stations for the four study areas of the Southeast Pacific.

Zone	Station	N	Latitude	Longitude	Date	Time	Depth (m)	Cruise	Sampling gear
CUP-Z	1	10	27°04′	70°57′	10–14–2016	23:13 (N)	0–100	CIMAR22	1 m^2^ Tucker trawl net
	4	2	27°00′	71°12′	10-15- 2016	08:51 (D)	0–1500	CIMAR22	8 m^2^ Tucker trawl net
	6	13	26°59′	72°38′	10-16- 2016	09:31 (D)	0–1600	CIMAR22	8 m^2^ Tucker trawl net
CTZ	16	7	25°15′	80°16′	10-25-2016	19:26 (N)	0–1000	CIMAR22	1 m^2^ Tucker trawl net
	17	3	26°15′	80°07′	10-23-2016	22:46 (N)	0–50	CIMAR22	1 m^2^ Tucker trawl net
	22	16	26°19′	79°56′	10-22-2016	21.45 (N)	0–150	CIMAR22	1 m^2^ Tucker trawl net
	23	1	29°22′	80°00′	11-02-2016	20:46 (N)	0–1000	CIMAR22	1 m^2^ Tucker trawl net
	24	2	30°33′	80°01′	11-03-2016	08:48 (D)	0–1000	CIMAR22	1 m^2^ Tucker trawl net
	25	4	31°47′	79°39′	11-04-2016	02:44 (N)	0–1000	CIMAR22	1 m^2^ Tucker trawl net
	34	1	33°35′	79°12′	11-04-2016	21:54 (N)	0–1000	CIMAR22	1 m^2^ Tucker trawl net
OLIGO-Z	13	14	26°59′	81°56′	10-20-2016	05:12 (N)	0–3000	CIMAR22	8 m^2^ Tucker trawl net
	18	10	27°01′	89°34′	10-21-2015	20:19 (N)	0–900	CIMAR21	1 m^2^ Tucker trawl net
U-OLIGO-Z	30	44	27°00′	107°34′	10-28-2015	02:29 (N)	0–900	CIMAR21	1 m^2^ Tucker trawl net
	64	5	26°33′	105°49′	11-01-2015	21:57 (N)	0–900	CIMAR21	1 m^2^ Tucker trawl net

Reported for each station are the number of *P. abdominalis* individuals (N), the longitude, latitude, date, time, sampling depth, cruise and the sampling gear. The oceanographic zones are specified as: eutrophic coastal upwelling zone (CUP-Z), mesotrophic coastal transition zone (CTZ), oligotrophic zone (OLIGO-Z) and ultra-oligotrophic zone (U-OLIGO-Z). Sampling time is specified as night (N) or day (D).

## Results

### Phylogenetic inferences

**Figure 2:**
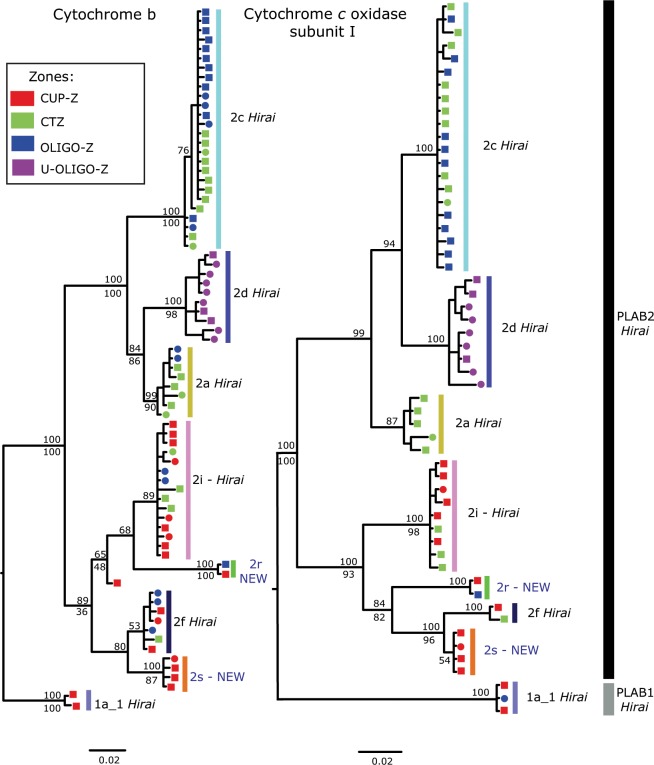
Bayesian phylogeny of *P. abdominalis* specimens from the Southeast Pacific inferred from partial sequences of the COI (right) and CytB (left) genes (55 COI, 75 CytB sequences). Numbers at the nodes indicate the Bayesian probability (above) and Maximum likelihood (below) support values. Mitochondrial clades are identified by letters (a-s) and colors. For each individual, the oceanographic zone of collection is indicated by its color (CUP-Z, CTZ, OLIGO-Z and U-OLIGO-Z; legend upper left). Individuals with both COI and CytB sequences included are indicated by squares, and individuals represented by a single sequence are marked by a circle. Clades identified with a ‘Hirai’ label are consistent with those reported in Hirai *et al*.^41^: NEW clades are first reported here.

**Figure 3:**
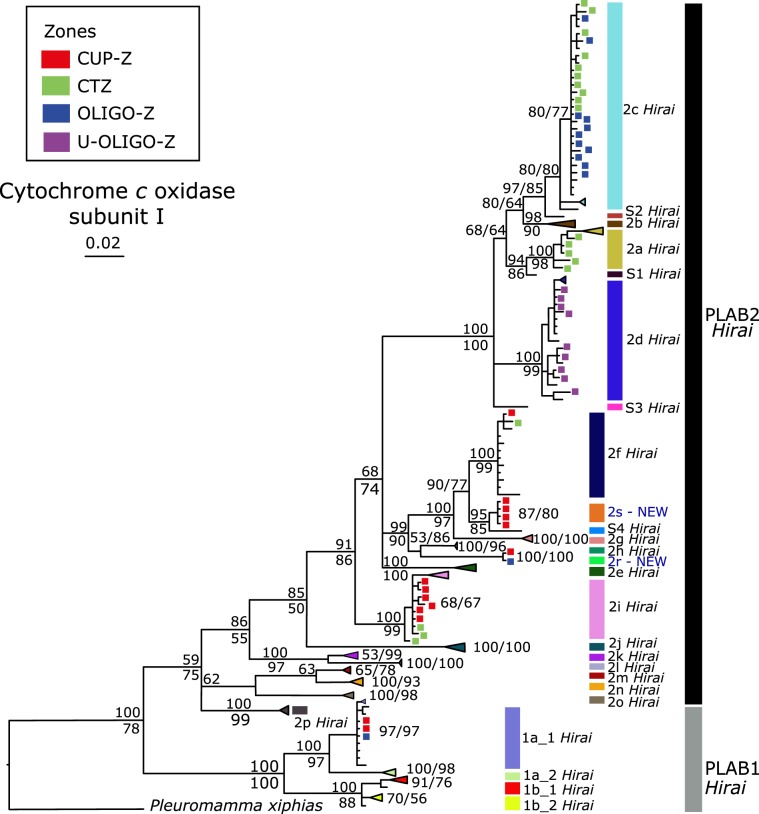
Bayesian phylogeny of 221 *P. abdominalis* COI sequences, including those from the Southeast Pacific reported in this study as well as 164 representatives from the global study of Hirai *et al*.^41^. Numbers at the nodes represent the Bayesian posterior probability (upper) and Maximum likelihood (lower) support values. Mitochondrial clades are labelled by letters (a-s) and colors. Accession numbers for the 164 *P. abdominalis* COI sequences from Hirai *et al*.^41^ are reported in Table S2. *P. xiphias* (accession number: JN574427) was used to root the tree. Higher level clades are labelled and referred to in the text (PLAB1, PLAB2).

Phylogenetic analyses of Southeast Pacific specimens using COI and CytB gene fragments resulted in trees that include eight primary clades within *P. abdominalis* (Fig. 2). Of the eight clades reported for the region, six were previously described by Hirai *et al*.^41^ (Fig. 3). In order to facilitate comparison of our results with those from other ocean regions, we identify the 8 clades present in the SEP by the clade identifiers originally reported by Hirai and authors (6 clades), and in the cases where the clades have not been previously detected, we give them a new identifier and flag them as new to the literature (e.g., 2r – NEW). Bayesian and Maximum likelihood phylogenetic analyses yielded concordant tree topologies.

The eight clades were split into two genetically very divergent clades, PLAB2 *Hirai* and PLAB1 *Hirai*, which showed high nodal support values [100% Maximum Likelihood (ML), 100% Bayesian Posterior Probability (BPP)] for both mitochondrial genes (Fig. 2). PLAB2 *Hirai* was more diverse than PLAB1 in the SEP and included seven clades (2a-2s) located across the four oceanographic study zones for the two mitochondrial markers. PLAB1 included only the clade 1a_1 *Hirai*, with haplotypes located mainly in the coastal upwelling zone. Within PLAB2 *Hirai*, seven clades were observed, which were divided into two large groups with high support values in both gene trees (100% ML, 100% BPP). The first group, including clades 2c *Hirai*, 2d *Hirai*, and 2a *Hirai*, showed variation in tree topology depending on the gene. In the CytB tree, clade 2c *Hirai* was sister to the pair of clades 2d *Hirai* and 2a *Hirai*, while for the COI gene, the clades 2c *Hirai* and 2d *Hirai* make up a group with a sister clade 2a *Hirai*. The second PLAB2 *Hirai* group, including four clades, also had high nodal support (CytB: 89% BPP, COI:100% BPP, 93% ML) and variation in topology depending on the gene. In the CytB tree we observed two groups, one formed by the clades 2i *Hirai* and 2r-NEW and another by the clades 2 f *Hirai* and 2s-NEW; however, in the COI tree the clade 2r-NEW was placed with clades 2 f *Hirai* and 2s-NEW.

Most of the eight primary clades were well supported as reciprocally monophyletic groups in both mitochondrial gene trees (>75% node support, ML/BPP). Nodal support values for each clade were high in Bayesian analyses (CytB: 53–100% BPP, COI: 54–100% BPP), with 6 or more clades with > 99% BPP support (both genes). ML bootstrap support values were lower, but still supported reciprocal monophyly (>75% ML) in the majority of clades in the CytB gene tree, with lower ML bootstrap values in the COI tree. In addition, placement of animals into clades was entirely congruent across the two mitochondrial trees, with animals present in both trees always occurring in the same mitochondrial clade (animals marked by a square in Fig. 2). In terms of composition, previously reported clades 2c *Hirai* and 2a *Hirai* mainly consisted of haplotypes from the transition and oligotrophic zones, while clade 2d *Hirai* included only individuals sampled in the ultra-oligotrophic zone. Clade 2 f *Hirai* consisted of individuals from the coastal upwelling and transition zones, and clade 1a_1 *Hirai* included specimens from the coastal upwelling zone and a single individual from the oligotrophic zone (COI). Finally, clade 2i *Hirai* was found in individuals distributed from the upwelling to the oligotrophic zones.

Two clades primarily sampled in the coastal upwelling and transition zones have not been found or reported in any prior work, and are newly described in this study: They are the clades 2r-NEW and 2s-NEW. Both were well supported as reciprocally monophyletic in Bayesian analyses (both genes, 100% BPP), with more moderate support in ML results, as was observed in the other 6 previously described clades. The clade 2r-NEW occurred in the coastal upwelling and oligotrophic zones, and clade 2s-NEW included only coastal upwelling zone animals. The eight *P. abdominalis* mitochondrial clades reported from the Southeast Pacific in the present study could be distinguished from other clades reported in a COI gene tree that included sequences from all *P. abdominalis* clades (globally), covering the equatorial divergence region, oligotrophic zones and even the coastal transition zone off South America (Fig. 3; Hirai *et al*. data)^41^. This analysis revealed that the sampling performed in this study included all clades reported for the South Pacific with the exception of the clades 2e *Hirai*, 2k *Hirai* and 2 m *Hirai*, which occur in the equatorial region (Fig. 3), and 1b-1 *Hirai* which occurs in the western South Pacific (see Suppl. Fig. S1 for global *P. abdominalis* phylogeny).

Genetic distances (D) calculated using the T92 substitution model for eight mitochondrial clades were higher for the CytB fragment (D = 0.056–0.495), with lower maximum genetic distances among clades observed at COI (D = 0.036–0.183) (Table 2). The highest genetic distance values for both genes were observed in comparisons between the clades PLAB1 and PLAB2. For CytB the highest values were observed between clades 1a_1 *Hirai* and 2r-New (D = 0.4952); however, for the COI gene, the maximum value was observed between the clades 1a_1 *Hirai* and 2c *Hirai* (D = 0.1831) (Table 2). Genetic distances observed between sister clades were generally between 0.04 and 0.07, for example between the clades 2s-NEW and 2 f *Hirai* (D < 0.056) and 2a *Hirai* with 2d *Hirai* (D < 0.0688), for both genes.

**Table 2:** Average T92 genetic distances among eight primary mitochondrial clades based on COI and CytB alignments.

	2c *Hirai*	2d *Hirai*	2a *Hirai*	2i *Hirai*	2r-New	2 f *Hirai*	2s-New	1a_1 *Hirai*
2c *Hirai*	—	0.044	0.052	0.121	0.135	0.138	0.132	0.183
2d *Hirai*	0.136	—	0.050	0.114	0.126	0.131	0.131	0.174
2a *Hirai*	0.095	0.069	—	0.106	0.125	0.127	0.124	0.170
2i *Hirai*	0.142	0.274	0.285	—	0.087	0.094	0.084	0.176
2r-New	0.419	0.443	0.414	0.142	—	0.093	0.088	0.173
2 f *Hirai*	0.271	0.199	0.195	0.138	0.153	—	0.036	0.176
2s- New	0.287	0.187	0.222	0.181	0.161	0.056	—	0.166
1a_1 *Hirai*	0.372	0.379	0.374	0.279	0.495	0.389	0.395	—

Distances for CytB are reported below the diagonal, and COI values are above the diagonal.

**Figure 4:**
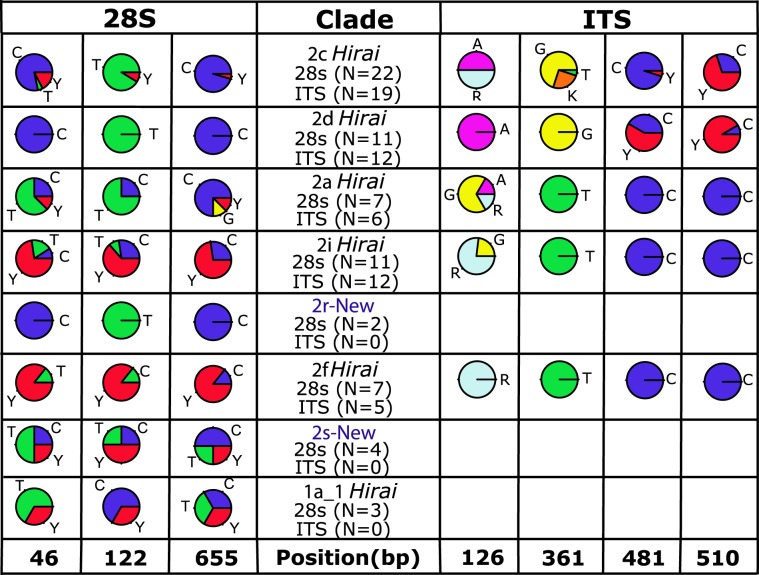
Nucleotide proportions at variable sites  for the fragments 28 S and ITS for each of the mitochondrial clades. Three polymorphic sites at positions 56, 122 and 655 are shown for the 28 S. In the ITS gene, four polymorphic sites were observed at positions 126, 361, 481 and 510. Alignments are available in Supplementary Files S1 and S2.

**Figure 5:**
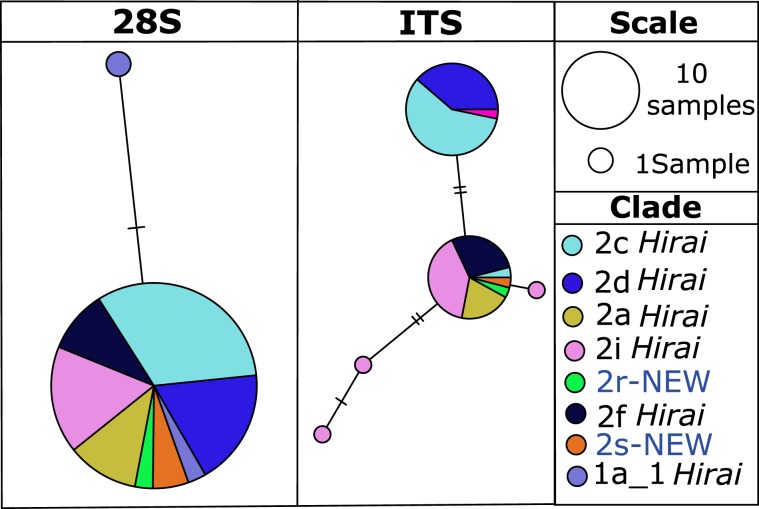
*P. abdominalis* statistical parsimony haplotype networks for  nuclear 28 S and ITS, with color indicating mitochondrial clades. Each hash mark represents a mutational step, and the size of the circles is proportional to the number of individuals sampled with that genotype.

The 28 S and ITS  sequences had low polymorphism, with 3 and 4 variable nucleotide sites in 28 S and ITS, respectively (Fig. 4). Analysis of the nuclear sequences for individuals for which we also have mitochondrial sequence found concordant genetic differences in both genomes between clades in many cases. Three informative sites were observed for the 28 S gene, located at bases 46, 122 and 655 (Fig. 4; Suppl. Alignments S1–S4). Focusing on clades with N > 5 animals sequenced at 28 S rRNA, analysis of these polymorphic sites revealed shifts in nucleotide composition between the closely-related clades 2c *Hirai*/2d *Hirai*/ 2a *Hirai* and 2i *Hirai*/2 f *Hirai*, with a higher frequency of Y at all 3 sites for the latter clade pair. The 28 S TCS haplotype network had an abundant central haplotype separated from another far less abundant haplotype that included only individuals belonging to clade 1a_1 *Hirai* (note that sites with ambiguous bases are masked in this analysis; Fig. 5).

Within the ITS fragment, we observed four polymorphic sites located at bases 126, 361, 481 and 510. Analysis of the relative frequency of the clades 2a *Hirai* and 2i *Hirai* revealed changes only in the first site, observing a change in the relative frequency of base R for the first clade with 16.7% and 76.9% for the second. On the other hand, within the clades 2d *Hirai* and 2c *Hirai* the penultimate site (481) showed changing frequency between the haplogroups, in the first clade a lower frequency of Y (5%) with the second one at 58.3%. Finally, clade 2 f *Hirai* was the only haplogroup with apparently fixed frequencies for all four polymorphic sites. The haplotype network for ITS revealed the presence of two abundant haplotypes separated by two mutational steps (Fig. 5). All animals from mitochondrial clade 2d *Hirai* and nearly all of the animals from closely-related 2c *Hirai* had one nuclear haplotype, while animals from the second major group of clades within PLAB2, including 2i *Hirai*, 2r-NEW, 2s-NEW, 2 f *Hirai*, all  were represented in the rest of the haplotype network (Fig. 5).

### Species delimitation

Two analytical approaches were used to delimit *Pleuromamma* species in our study region, the Generalized Mixed Yule Coalescent (GMYC) likelihood method and the Poisson Tree Process (PTP) model, using single marker gene trees for both COI and CytB^42^. GMYC and PTP analyses for both mitochondrial genes supported the inference of several species being present within the nominal species *P. abdominalis*. In the GMYC results, the number of distinct entities (clusters) associated with the highest likelihood score for the COI gene was 7 (confidence interval 7–9; *p* < 0.001), with 9 entities identified for CytB (confidence interval 8–9; *p* < 0.001). GMYC support values for most clades were high at both mitochondrial genes (>0.6; Table 3), confirming the phylogenetic results reported above. The groups identified by GMYC analyses were concordant with the eight mitochondrial clades previously described (Fig. 2). Clade 2 f *Hirai* had relatively low support (0.11) at the COI gene, but was well supported by analyses of CytB. PTP analysis indicated the presence of 11 species within the study sequences for both COI and CytB genes. This analysis showed concordance with the six mitochondrial clades described above with high support values (>0.5 BPP). However, PTP analyses also indicated greater splitting, for example both mitochondrial genes indicated that clade 2d *Hirai* was composed of 3 species and clade 2c *Hirai* by 2 species. Because greater congruence was observed between GMYC and phylogenetic results (above), we place greater emphasis on these inferences.

**Table 3:** GMYC support values for each mitochondrial clade based on COI and CytB gene fragments.

Support values from GMYC analyses			
Clade	Zone	COI	CytB
2c *Hirai*	OLIGO-ZCTZ	0.77	0.88
2d *Hirai*	U-OLIGO-Z	0.66	0.95
2a *Hirai*	OLIGO-ZCTZ	0.89	0.97
2i *Hirai*	CUP-ZCTZOLIGO-Z	0.88	0.97
2r-NEW	OLIGO-ZCTZ	0.98	1.00
2 f *Hirai*	CUP-ZCTZ	0.11	0.74
2s-NEW	CUP-Z	0.89	0.77
1a_1 *Hirai*	CUP-ZOLIGO-Z	1.00	1.00

Clades with support values closer to 1.0 have a higher probability of being a distinct, currently undescribed species. Oceanographic zones as described in Table 1 and Fig. 1.

### Oceanic biogeography of mitochondrial clades

**Figure 6:**
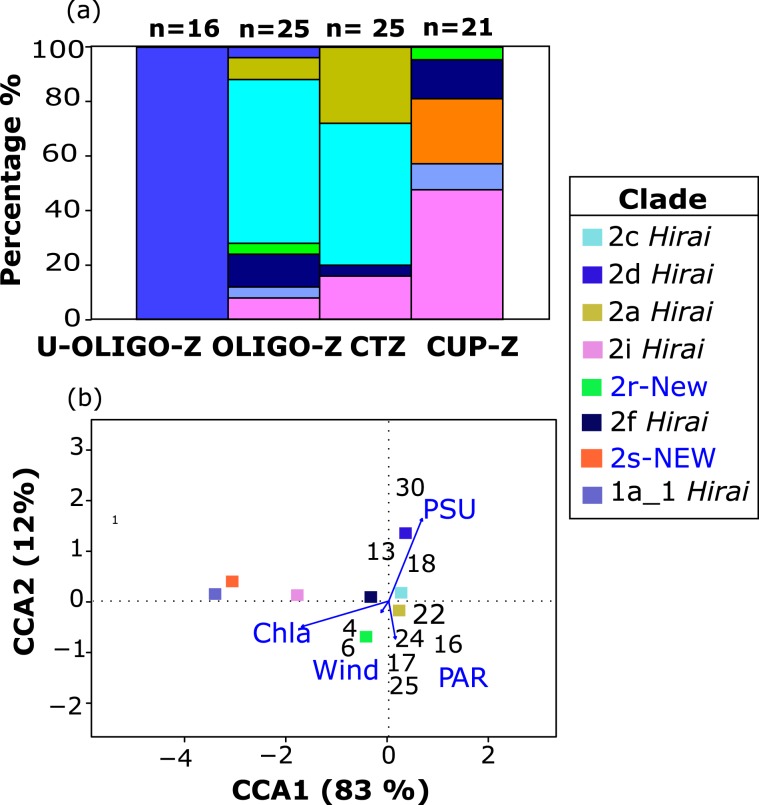
Oceanographic distribution of mitochondrial clades and their correlation with hydrographic variables in the Southeast Pacific. (**a**) Variation in relative abundance (%) of mitochondrial clades observed from the Chilean coast (right) to Easter Island (left), (**b**) Biplot of CCA results for mitochondrial clades and hydrographic variables at each station. Results shown for the most parsimonious model (see Suppl. Table S1). Length of the arrows indicates the correlation between hydrographic variables and ordination axes. Numbers within the diagram represent the different sampling stations where individuals were collected.

When analyzing *P. abdominalis* mitochondrial clades present in the Southeast Pacific, high variability was found in their relative abundance across the four study zones (Fig. 6a). In the ultra-oligotrophic zone near Easter Island, all animals sampled were from clade 2d *Hirai* (100% relative abundance). In the oligotrophic and transition zones, four clades were observed, including 2a *Hirai*, 2c *Hirai*, 2 f *Hirai* and 2i *Hirai*. Within these two regions, clade 2c *Hirai* showed the highest relative abundance (>50%) with a second clade 2a *Hirai* also occurring at high frequency (>8%). Within the oligotrophic zone, three clades occurred with low relative abundance (<5%) that were not shared with the transition zone, including clade 2d *Hirai*, with overlapping distribution in the ultra-oligotrophic zone, and 2r-New and 1a_1 *Hirai* that also were present in the coastal upwelling zone. The coastal upwelling zone was dominated by the mitochondrial clades 2i *Hirai* (>50%) and the newly discovered 2s-NEW (<30%), the latter being exclusive to this zone. Some haplogroups showed a decrease in their relative abundance from the upwelling to oligotrophic zones as observed in clade 2i *Hirai* and 1a_1 *Hirai*; however, others remained relatively constant across zones, as in the rarer clades 2 f *Hirai* and 2r-NEW (Fig. 6a).

The CCA between abundance of the eight mitochondrial clades and the hydrographic variables indicated that the most parsimonious and significant model (*p-value* < 0.05), comprised of chlorophyll-a (Chla), sea surface salinity (SSS), wind stress (WT) and photosynthetically active radiation (PAR), explained 94% of the variability (Suppl. Table S1). The first canonical axis (CCA1) captured 83% of the variance and covaried primarily with Chla, while the second axis (CCA2) explained 12% of the variance and was primarily capturing variation in salinity. The clades located in the coastal upwelling zone,1a_1 *Hirai*, 2s-NEW and 2i *Hirai*, were observed in regions of high Chla, showing a strong negative correlation with the CCA1 axis. Clades 2a *Hirai*, 2c *Hirai* and 2 f *Hirai*, which occurred across the coastal transition and oligotrophic zones, were located near 0.0 of the CCA plot indicating that the hydrographic variables of the model were not good predictors of the distribution of these clades, suggesting less dependency on the hydrographic regime. These clades were observed in environments that were intermediate or variable in hydrographic conditions (Chla, Salinity). Finally, clade 2d *Hirai*, the only clade present in the ultra-oligotrophic zone, showed strong association with high SSS environments and had a strong positive correlation with the CCA2 axis (high salinity, low Chla) (Fig. 6b).

## Discussion

Molecular analysis in zooplankton species with cosmopolitan distributions has revealed a variable number of genetic lineages in organisms that were ascribed as single species using traditional taxonomy^24,43,44^. These distinct lineages commonly exhibit patterns of distribution associated with variable oceanographic conditions^22^. Our study constitutes a first step to disentangle the genetic diversity within *P. abdominalis* in the Southeast Pacific. Phylogenetic analysis of mitochondrial and nuclear genes of the single species *P. abdominalis* showed a complex of genetic entities not described morphologically, suggesting a process of cryptic or pseudocryptic speciation. To assess this possibility, we delimited putative species using a Generalized Mixed Yule Coalescent approach, finding that these 8 clades likely correspond to different species. Subsequently, we investigated the distribution of these cryptic or pseudocryptic species and found them linked to oceanographic variability. Phylogenetic analysis of COI and CytB gene fragments indicated this nominal species as having high diversity over a rather limited geographic range (4000 km), in comparison with some other oceanic copepods of cosmopolitan distribution^44,45^. The different mitochondrial lineages observed within *P. abdominalis* may thus represent distinct species^41^, or ancient mitochondrial lineages that persist within large populations of oceanic plankton. The *P. abdominalis* lineages detected and reported here extend the distribution of material examined in Hirai *et al*.^41^, and suggest that a number of additional genetic lineages within the nominal species may yet be found across the circumglobal distribution of this species.

Our study further suggests that different clades are adapted to specific oceanographic conditions. Some mitochondrial lineages showed a wide distribution in the SEP, from the upwelling zone to the oligotrophic gyre (e.g. clades 2 f *Hirai*, 2r-NEW, 2i *Hirai*, 1a_1 *Hirai*) (Fig. 2). In contrast, other clades had a more limited distribution within the coastal transition and oligotrophic zones (e.g. clades 2a *Hirai*, 2c *Hirai*), while the remaining clades showed an unexpectedly high level of endemism: one in the ultra-oligotrophic zone (clade 2d *Hirai*), and the other in the coastal upwelling zone (clade 2s-NEW) (Fig. 2). The latter represents a new undescribed lineage of *P. abdominalis*, comparable to other endemic species of zooplankton known from the Humboldt Current, such as the calanoid copepod *Calanus chilensis* and euphausiid *Euphausia mucronata*, both of which are restricted to the upwelling zone^20,21^. Our phylogenetic analysis included all lineages already reported for distinct regions of the South Pacific and therefore it became clear that our two new clades had not been described previously, reflecting a hidden speciation process within the SEP basin.

In general terms, the genus *Pleuromamma* is considered omnivorous and this generalist feeding behavior may allow a wider spatial distribution^46^. However, we observed that the 8 clades were highly correlated with Chla and salinity allowing us to establish distributional limits (Fig. 6, Suppl. Table S1). The lineages found primarily within the coastal upwelling and in some cases extending into the transition and oligotrophic zones were generally distributed in areas with higher Chla (e.g., 2i *Hirai*), and these clades are possibly associated with nutrient-rich waters in the coastal upwelling zone and also in oligotrophic areas subjected to mesoscale eddies that can transport upwelled waters from the coastal zone into the oceanic region^47,48^. The clade 2i *Hirai* was previously described as widely distributed in the upwelling equatorial region Hirai *et al*.,^41^ and therefore its presence in the Humboldt Current indicates an extension of the described range towards upwelling areas of the SEP, linked to high levels of Chla.

On the other hand, the lineages primarily within the coastal transition and oligotrophic zones exhibited a correlation to low Chla areas (e.g., 2c *Hirai*), and they might also be associated with other oceanographic factors not considered in our study. Weak correlations with the predictors of our study suggest this may be the case. Finally, the lineage located in the ultra-oligotrophic zone showed strong correlation to high salinity (2d *Hirai*), which is likely a water mass tracer for the subtropical gyre (Fig. 6b). Hirai *et al*.^41^ also reports this lineage extending to the west of our sampling domain, but not beyond the ultra-oligotrophic conditions of the eastern limb of the South Pacific Subtropical Gyre. Adaptation to specific ranges of temperature, salinity, or other oceanographic parameters has been reported as an important driver for genetic diversification in several planktonic organisms, such as chaetognaths and calanoid copepods^49,50^, jellyfishes^51^, and estuarine copepods^52^.

The high level of endemism found, in particular in the upwelling and in the ultra-oligotrophic zones, may be a response to both present and historical physical processes. In the pelagic environment there are several physical barriers, such as land masses and oceanic currents, that can influence dispersal and connectivity among populations and act to promote species diversity^28,43,53–55^. The upwelling zone shows strong interactions among oceanographic forces, for example upwelling fronts, eddies, and tidal currents. These interactions may exert subtle effects on pelagic organisms over short time scales, but over large scales they may have profound effects on population structure^56–58^. For instance, the ultra-oligotrophic zone has been described as a region where populations exhibit different genetic composition^28,59,60^. This is because the subtropical gyres act as retention areas, and so allow genetic structuring to develop and persist^27–29,41,59,61^. Over longer time scales, it has been suggested that during the last maximum glacial (LGM, 19,000 to 23,000 years ago) the productivity in the coastal zone was much higher^62^, while the subtropical gyre had lower productivity^63^. This contrast may have acted as an allopatric barrier for dispersal between the zones, explaining the present divergence of the ultra-oligotrophic area (clade 2d *Hirai*) with respect to the other zones. Later, by the end of the Pleistocene, warming of surface seawater, sea level rise, and lowering of productivity in the coastal zone may have weakened the oceanographic gradient and dispersal barriers between the oligotrophic and upwelling zones, and thus allowing an expansion and possibly secondary contact between zones generating the current parapatric pattern^11,64^. In any case, it has been well documented that distributional ranges can change rapidly, as evidenced presently due to climate change (e.g.  range shifts of ~23 km/year)^65^.

The GMYC analyses of mitochondrial genes clearly indicated that *P. abdominalis* is likely a complex of species comprised of genetically divergent clades that are reproductively isolated but difficult to distinguish morphologically. Our results suggest the presence of at least 7 species, within which two appear highly specialized in two particular zones, the upwelling and the ultra-oligotrophic ones. Other studies focused on cosmopolitan species within the same genus, such as *P. xiphias, P. gracilis*, and/or *P. piseki*, also have described the presence of several lineages, suggesting that a number of species in this genus are in fact cryptic species complexes^29,44^. The prior study by Hirai *et al*.^41^ inferred with certainty that *P. abdominalis* was comprised of at least two species coinciding with the clades PLAB1 and PLAB2, given concordant patterns of genetic variation between mitochondrial and nuclear markers. However, there was an open possibility for many more species, because the observed divergence within both clades was greater than 4.3%^41^. In the present study, values of divergence greater than 30% were reported among the 8 clades, yet both mitochondrial genes also showed that the greatest genetic distances were observed among the clades PLAB2 and PLAB1. In general terms, many described species of marine copepods exhibit minimum COI divergences  to sister species within the range of 8 to 9%^66,67^, while other work suggests that intraspecific variation of copepod lineages occurs within the range of 1 and 4%^68,69^.

The 28 S and ITS  sequences were less polymorphic and less informative than mitochondrial genes in detecting species limits within *P. abdominalis*. In the haplotype networks, both nuclear genes failed to show clear patterns with respect to the pre-established mitochondrial clades (Fig. 5). However, these  sequences showed differences in nucleotide frequencies at polymorphic sites, allowing us to distinguish several of the 8 clades by means of the percentage of the nucleotide bases (Fig. 4). The lack of clear concordance between our mitochondrial and  nuclear  markers can be explained by greater sensitivity of mitochondrial markers to genetic drift than nuclear markers, due to haploid uniparental heredity and smaller effective population size^70–72^. Work conducted in other copepod species has reported that the 28 S gene shows a slower rate of evolution than that of the COI gene^54,73,74^ and incomplete lineage sorting may occur. Furthermore, it is well known that in order to effectively delimit species it is necessary to analyze multiple loci^52,75–77^. This is because single locus analysis can either underestimate or overestimate the number of species due to the presence of pseudogenes^78^, ancient mitochondrial lineages that are present within interbreeding populations (single species), and introgression among closely related species as well as other processes that may obscure species boundaries. Several studies have shown that the use of mitochondrial markers may be more efficient for initially detecting cryptic species^45^, with subsequent studies using a combination of markers for consistent species delimitation (e.g., copepod *Haloptilus longicornis*)^43^. In any case, the high level of genetic diversity and endemism observed here seems unexpected within an ocean basin that is subject to both meridional and zonal advection and water mixing, suggesting that evolution and speciation processes can occur more rapidly than traditionally thought in the open ocean for short-lived species like pelagic copepods.

## Methods

### Oceanographic survey and samples

Zooplankton samples were collected during two oceanographic cruises conducted in the Southeast Pacific (Fig. 1, Table 1). The first cruise, CIMAR-21, sampled during 01–30 October 2015 and the second cruise, CIMAR-22, from 14 October to 13 November 2016. The first cruise focused on a transect between Caldera (27°S, 70°50′W) and Easter Island (27°10′S, 109°30′W), covering a total of 30 oceanographic stations. The second cruise sampled 25 oceanographic stations along two zonal transects at 26.5°S and 33°S, and one meridional transect, between the Desventuradas Islands and the Juan Fernández Archipelago.

The cruises spanned a large oceanographic gradient, and we differentiated the study regions using satellite-derived Chla, with observations during the two cruise periods (https://coastwatch.pfeg.noaa.gov/erddap/index.html). We differentiated four zones according to the observed near-surface Chla ranges: a eutrophic zone corresponding to the coastal upwelling region of Chile with Chla > 0.5 mg m^−3^ (CUP-Z), the mesotrophic coastal transition zone (CTZ) that shows a range of Chla between 0.1–0.5 mg m^−3^, the oligotrophic zone (OLIGO-Z) with variation between 0.05–0.10 mg m^−3^, and finally an ultraoligotrophic zone (U-OLIGO-Z) that is located in the central region of the South Pacific gyre with concentrations of Chla < 0.05 mg m^−3^ (as shown in Fig. 1). Similar zonation has been reported in previous works^5,11,12^.

 Specimens were collected at a total of 13  stations during the CIMAR-21 and CIMAR-22 cruises: 25 within the CUP-Z, 34 in the CTZ, 24 in OLIGO-Z and 49 in U-OLIGO-Z (Table 1). Mesozooplankton were collected with a 1 m^2^ Tucker trawl (200 μm mesh) on both cruises, and an 8 m^2^ Tucker trawl net (300 μm mesh size) on CIMAR-22 (Table 1). Most samples were collected at night and covered a wide depth strata (>100 m in most cases). The number of specimens per station, coordinates of the sampling sites, date, time, maximum depth of the plankton net, cruise and gear used at each station are reported in Table 1. Plankton samples for genetic analysis of the four study zones were frozen at −20 °C at sea. In the laboratory, samples were thawed and screened with a 200 μm sieve to eliminate excess water and were subsequently preserved with 95% ethanol. The ethanol was replaced within 24 hours of initial preservation and samples subsequently stored at −20 °C. Adult female specimens were sorted from bulk plankton samples and identified as *P. abdominalis* with the aid of taxonomic keys by Bradford-Grieve^38^. All specimens of the target species found in the CUP-Z and CTZ areas were selected; however, for the most oceanic areas (OLIGO-Z and U-OLIGO-Z), with greater abundance of the target species, individuals were chosen randomly.

### DNA extraction and polymerase chain reaction (PCR) amplification

Specimens selected for genetic analysis were transferred to an Eppendorf tube with 1 ml of MilliQ water, and incubated at room temperature for 12 hours, in order to eliminate any remaining ethanol. Genomic DNA from specimens collected on the CIMARR 22 cruise were extracted with the Mollusc DNA Kit (Omega) following manufacturer protocols. Specimens collected on CIMAR 21 were extracted using the Forensic DNA Kit (Omega) following manufacturer protocols, with the exception of incubating the sample with the lysis buffer and proteinase K for only 1 hour. Extracted DNA was maintained at −20 °C.

Fragments of the genes encoding for mitochondrial proteins cytochrome *c* oxidase subunit I (COI) and cytochrome *b* (CytB) as well as nuclear genes encoding the large subunit of the ribosome (28 S) and internal transcribed spacer regions (ITS1-5.8SrDNA-ITS2) were used for phylogenetic analyses. The primers used to amplify each gene fragment, the length of the amplified fragment, and the annealing temperatures used during PCR amplification are specified in Table 4. PCR amplifications were performed in a total volume of 28 μl for 35 cycles, including 4 μl of 5X Green GoTaq G2 Flexi Buffer, 4.48 μl of 25Mm MgCl_2_ 25, 2 μl of dNTP (final concentration of each 0.25 Mm), 2 μl of each primer (0.35 mM), 0.045 units of the GoTaq G2 Flexi DNA Polymerase (Promega) and 5 μl of the DNA template. PCR products were visualized by 1% agarose gel electrophoresis and sent to Macrogen for sequencing. Sequencing of PCR products was performed primarily along the sense (forward) strand; both strands were sequenced if the forward reads were ambiguous at some nucleotide sites.

**Table 4:** Names of the primers used, sequences [5′ to 3′], literature sources and the annealing temperature used in PCR.

Fragment	Primer name, sequence	Product (Alignment)	AT
COI	COI (F), GGTCATGTAATCATAAAGATATTGG^74^	~800 bp (639 bp)	47 °C
	PLAB-R(R), GTKGTAAAATATGCYCGTGTGTC^41^		
CytB	UCYTB151F, TGTGGRGCNACYGTWATYACTAA^100^	~350 bp (281 bp)	45 °C
	UCYTB270R, AANAGGAARTAYCAYTCNGGYTG^28^		
28 S	LSU Cop-D1F, GCGGAGGAAAAGAAAACAAC^101^	~800 bp (754 bp)	55 °C
	LSU Cop-D3R, CGATTAGTCTTTCGCCCCT^28^		
ITS	LR1(F), GGTTGGTTTCTTTTCCT^102^	~800 bp (687 bp)	50 °C
	SR6R (R), AAGWAAAAGTCGTAACAAGG^103^.		

The expected length of the PCR product in base pairs (bp) for each gene fragment is indicated, together with the length of the alignments used in phylogenetic analyses (in parentheses).

### Sequence alignment and phylogenetic analyses

Sequences from both strands of the same specimen were aligned, base calls confirmed and edited manually, and consensus sequences produced using Geneious 7.1.3^79^. A BLAST search was conducted on confirmed and consensus sequences in order to verify the absence of contamination (correct organism)^80^. CytB sequence amino acid translations contained no stop codons. Within COI, a total of 6 individuals were found to have a 1-base indel, which would result in a frame-shift mutation. These sequences were inferred to derive from a COI pseudogene and these individuals were removed from all analyses. All included animals have COI sequence translations with no stop codons. Final alignments were created for each gene fragment using MUSCLE within Geneious 7.1.3^79^. A total of 55 sequences were aligned for COI, 75 for CytB, 102 for 28 S rRNA and 80 for ITS. The length of the alignment obtained for each gene fragment is reported in Table 4 (Suppl. Alignments S1-S4).

Phylogenetic analyses were performed using both maximum likelihood (ML) and Bayesian inference (BI) methods for both mitochondrial genes. The T92 + I and T92 + G models were selected as the best-fit nucleotide substitution models by the Bayesian Information Criterion (BIC), as performed in Mega-X V.10^81^ for COI and CytB, respectively. Bayesian analyses were carried out in MrBayes V.3.1^82^. The Markov Chain Monte Carlo chains were executed in three simultaneous runs with default heating, selected the appropriate models of sequence evolution obtained from MEGA-X V.10^81^, over 1.0 × 10^6^ generations, and trees were sampled every 1000 generations for both mitochondrial genes. The first 25% of the trees generated were eliminated (as burn-in). Stationarity was estimated by standard deviation of the three runs (>0.001) and by observation of stationarity when the curve of the log-likelihood scores against generation time in Tracer was non-trending^83^. Maximum likelihood analyses were performed with the GTR + G model and with 5000 bootstrap replicates to calculate the support for each node using RAxML V.8.2.10^84^. ML and BI trees were inferred for all newly obtained sequences from the SEP for both COI and CytB gene fragments (separately), as well as including representative COI sequences from clades reported in Hirai *et al*.^41^. In the latter tree, the analysis included 164 sequences representing all clades of *P. abdominalis* from the global Hirai *et al*.,^41^ study (GenBank access numbers as indicated in Suppl. Table S2), in addition to the 54 sequences newly reported here. A global tree for *P. abdominalis* also was inferred with 944 sequences from Hirai *et al*., 2015 [GenBank accessions: KT319926.1- KT320869.1^41^] and including sequences new to this study, in order to assign mitochondrial clades in the present study to existing names or identify them as new lineages that had not been previously reported. We included COI sequence from the sympatric species *P. xiphias* [GenBank access numbers: JN574427^29^] as an outgroup in phylogenetic analyses.

We used Popart V 1.7^85^ to create a graph presentation of a TCS network for the 28 S and ITS fragments, which illustrates the relationship between nuclear haplotypes and mitochondrial clades^86^. In these TCS networks, nucleotide sites with > 5% ambiguities were masked (not included). In doing this, in the case of 28 S, much of the observed variation was thus masked. Genetic distances between mitochondrial clades were calculated as the net average between clades using final alignments for each gene, with 10,000 bootstrap replicates, using the Tamura 3-parameter (T92) nucleotide substitution model (Tamura, 92) and uniform rates between sites for COI and gamma-distributed rate variation for CytB (using Mega-X V.10^81^).

### Species delimitation

Two analytical approaches were used to delimit *Pleuromamma* species in our study region, the Generalized Mixed Yule Coalescent (GMYC) likelihood method and the Poisson Tree Process (PTP) model. The GYMC analysis is a likelihood method that enables delimitation of the number of species based on model fitting the branching patterns expected within and between species^87^. We inferred ultrameric gene trees for the COI and CytB alignments using BEAST 2.0^88^. Parameters selected for analysis included the HKY nucleotide substitution model, 6 categories for gamma-distributed rate variation, a relaxed lognormal clock, and a Yule model to express the branching pattern of the tree for both genes. Two independent runs were performed to ensure convergence, each run for 50 million generations and with sampling every 10,000 generations. Output files were visualized in Tracer v1.6^83^ to ensure convergence of the chains. The maximum clade credibility (MCC) tree was determined by Tree Annotator v1.8^89^ after removing the first 25% of trees (as a burnin).The number of delimited species was inferred for each MCC gene tree using the “gmyc” function in the splits R package^90^, with the single threshold option selected, as recommended by Fujisawa and Barraclough^87^. The GMYC analysis compares a null model (without a threshold) and an alternative hypothesis (with a threshold) in order to evaluate whether all samples constitute a single species (null) against the alternative hypothesis of the existence of more than one species in the data. A likelihood ratio test (LRT) is used to evaluate the model results, with significance calculated using the chi-square test with two degrees of freedom. The analysis also gives the number of genetic entities with their confidence intervals (IC), and the sequences that are associated with each group.

We also performed a PTP delimitation of species using the bPTP web server http://species.h-its.org/ptp/. This model uses coalescence theory and estimates the speciation rate from the number of substitutions observed across the tree. As the input phylogeny, we used the COI and CytB Bayesian phylogenies. We ran the PTP analysis with 5 × 10^5^ generations of MCMC sampling, with a thinning value of 100 and a burnin of 25%. MCMC convergence was visually confirmed as recommended by Zhang *et al*.^91^.

### Biogeography

A canonical correlation analysis (CCA^92^) was performed to assess the association between hydrographic variables and mitochondrial clades occurring at each station. The model was constructed with eight response variables corresponding to the eight genetic clades, and as predictor variables, we selected sea surface values of chlorophyll-a (Chla), temperature (SST), photosynthetically active radiation (PAR), salinity (SSS), wind stress (WT) at the sea surface, an upwelling index (UI), which is an estimate of water mass being transported offshore by the alongshore wind^93^, and wind stress curl (WSC) representing the curvature of the tension of the surface wind^94^. CCA analysis incorporates both ordination and multiple regression techniques for the analysis of relationships among multivariate data, and can be an efficient tool to relate categorical and environmental predictive variables^95–97^. Satellite data were used to describe oceanographic conditions during the CIMAR 21 and CIMAR 22 cruises. For each sampling station, the average value was calculated over three days, from one day before to one day after sampling, with a spatial resolution of 0.025 degrees (Table 1). Chla, SST and PAR values were obtained from the MODIS satellite (https://podaac.jpl.nasa.gov/datasetlist?search=MODIS),WT was obtained by a sensor aboard the EUMETSAT METOP satellite (https://manati.star.nesdis.noaa.gov/products/ASCAT.php), SSS was downloaded from the SMOS radio telescope (https://earth.esa.int/web/guest/missions/esa-operational-eo-missions/smos), and finally values of UI and WSC were obtained by the instrument Advanced Scatterometer (ASCAT) from NOAA Coast Watch (https://data.noaa.gov/dataset/dataset/wind-stress-metop-ascat-0-25-degrees-global-near-real-time-curl3).

The CCA was performed using the CCA package in Rstudio V.3.5^98^. To determine which variables to include in this analysis, a correlation analysis among the hydrographic variables was carried out. SST was found to have high correlation (>62%, *p-value* < 0.05) with Chla and salinity, and it was therefore eliminated from the analysis in order to minimize collinearity of hydrographic variables. The CCA was conducted with two contingency tables based on 1000 Monte Carlo permutations: one (X) represented the number of animals within each mitochondrial clade observed at each station and the second (Y) included values of Chla, SSS, WT, IU, WSC and PAR values for each station. The hydrographic variables that explained the largest component of variation in the distribution of the different mitochondrial clades were used to calculate the two most parsimonious canonical components. To evaluate significance of the most parsimonious model the Wilks’ lambda likelihood test was used, which indicates the variance not accounted for by the independent variables, and chi-square tests were used to evaluate the significance of lambda^99^.

## Supplementary information

Supplementary information.

Supplementary information.

## Data Availability

Sequence data reported in this publication are available through NCBI under accession numbers: MN045402 - MN045456 (COI), MN045457- MN045531 (CytB), MN069652 - MN069731 (ITS), and MN069732 - MN069833 (28 S rRNA). Alignments of the nuclear sequences are available in Supplementary Alignments S1 and S2.
