# Supplementary material for: Genetic diversity and novel lineages in the cosmopolitan copepod Pleuromamma abdominalis in the Southeast Pacific
Source: Sci Rep. 2020 Jan 24;10:1115. doi: 10.1038/s41598-019-56935-5 (PMC6981114; doi:10.1038/s41598-019-56935-5)

Supplementary Information

Genetic diversity and novel lineages in the cosmopolitan copepod *Pleuromamma abdominalis* in the Southeast Pacific

Carolina E. González, Erica Goetze, Rubén Escribano, Osvaldo Ulloa and Pedro Victoriano.

Supplementary table S1. Results of Canonical Correlation Analysis (CCA) for mitochondrial clades and hydrographic variables. Correlation of ordination axes with variables, eigenvalues and percentage variances explained. Significance of the permutation test for CCA between genetic and hydrographic variables as indicated (*p-value*). The hydrographic variables which contributed to the most parsimonious model were surface chlorophyll-a (Chla), sea surface temperature (SST), surface salinity (SSS) and wind stress (WT)

Variables	Axis 1	Axis2	*p-value*
Chla	**-0.9547**	-0.281	0.009***
PAR	0.0737	-0.414	
SSS	0.3662	**0.903**	
WT	-0.0833	-0.124	
Cumulative percentage of variance explained	0.8267	0.943	

Supplementary Table S2. Accession numbers for 164 *P. abdominalis* COI sequences globally from GenBank indicated by Hirai *et al.,* 2015 [41].

Clade	Access numbers
1b_2 *Hirai*	KT320131.1, KT320109.1, KT320109.1, KT320098.1, KT320082.1, KT320081.1, KT320080.1, KT320106.1, KT320104.1, KT320095.1
1b_1 *Hirai*	KT320516.1, KT320672.1, KT320645.1, KT320643.1, KT320543.1, KT320514.1, KT320504.1, KT320491.1, KT320512.1, KT320497.1
1a_2 *Hirai*	KT320253.1, KT320245.1, KT320243.1, KT320252.1, KT320241.1, KT320261.1, KT320260.1, KT320258.1, KT320244.1, KT320242.1
1a_1 *Hirai*	KT320767.1, KT320766.1, KT320764.1, KT320759.1, KT320765.1, KT320762.1, KT320763.1, KT320761.1, KT320760.1, KT320758.1
2p *Hirai*	KT320600.1, KT320553.1, KT320539.1, KT320500.1, KT320486.1, KT320466.1, KT320465.1, KT320414.1, KT320410.1, KT320208.1
2o *Hirai*	KT320588.1, KT320562.1
2n *Hirai*	KT320380.1, KT320378.1, KT320375.1
2m *Hirai*	KT320626.1, KT320398.1, KT320397.1, KT320396.1, KT320395.1, KT320393.1, KT320390.1, KT320389.1
2l *Hirai*	KT320309.1, KT320145.1, KT320129.1, KT320124.1
2k *Hirai*	KT320743.1, KT320736.1, KT320717.1, KT320716.1, KT320715.1, KT320712.1, KT320702.1
2j *Hirai*	KT320294.1, KT320297.1, KT320284.1, KT320283.1, KT320282.1, KT320281.1, KT320280.1, KT320278.1, KT320285.1, KT320279.1
2i *Hirai*	KT320327.1, KT320316.1, KT320349.1, KT320346.1, KT320329.1, KT320321.1, KT320345.1, KT320344.1, KT320355.1, KT320319.1
2e *Hirai*	KT320356.1, KT320352.1, KT320351.1, KT320350.1, KT320328.1, KT320220.1, KT320354.1, KT320353.1, KT320348.1, KT320347.1
2h *Hirai*	KT320488.1, KT320416.1, KT320412.1
2g *Hirai*	KT320219.1, KT320038.1
S4 *Hirai*	KT320303.1
2f *Hirai*	KT320705.1, KT320699.1, KT320698.1, KT320690.1, KT320689.1, KT320683.1, KT320680.1, KT320678.1, KT320619.1, KT320704.1

S3 *Hirai*	KT320566.1
2d *Hirai*	KT320775.1, KT320768.1, KT320770.1, KT320772.1, KT320773.1, KT320771.1, KT320769.1, KT320774.1, KT320714.1, KT320711.1
S1 *Hirai*	KT320540.1
2a *Hirai*	KT319935.1, KT319933.1, KT319931.1, KT319927.1, KT319926.1, KT319930.1, KT319928.1, KT319936.1, KT319932.1, KT319929.1
2b *Hirai*	KT320039.1, KT320027.1, KT320023.1, KT320016.1, KT320020.1, KT320017.1, KT320036.1, KT320022.1, KT320035.1, KT320011.1
S2 *Hirai*	KT320485.1
2c *Hirai*	KT320418.1, KT320555.1, KT320550.1, KT320533.1, KT320655.1, KT320651.1, KT320649.1, KT320646.1, KT320652.1, KT320641.1

Supplementary figure S1. Bayesian phylogeny of 999 *P. abdominalis* COI sequences reported in this study and at level global in Hirai et al., (2015). Numbers at the nodes represent the Bayesian posterior probability (upper) and maximum likelihood (lower) support values. Mitochondrial clades are labelled by letters (a-s) and colors. The tree includes 944 *P. abdominalis* COI sequences from GenBank (accession numbers: KT319926.1- KT320869.1; Hirai et al., 2015) and also a *P. xiphias* sequence (access numbers: JN574427) that was used to root the tree. Higher level clades are labelled and referred to in the text (PLAB1, PLAB2).

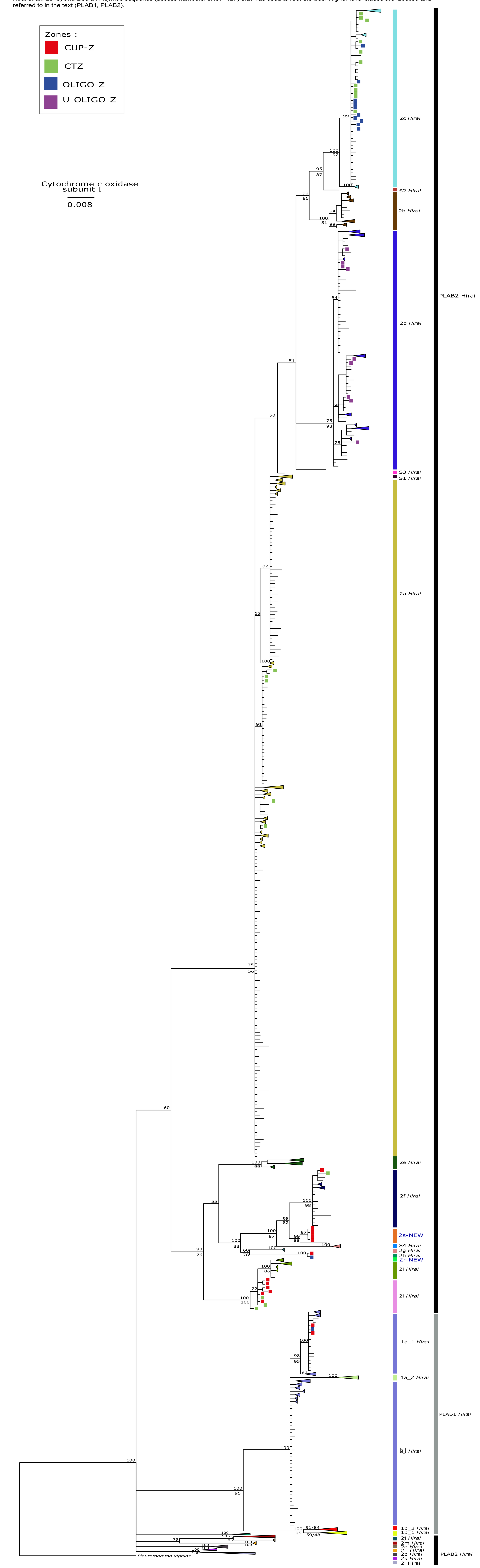

Supplement: Supplementary file 1 — Supplementary information. [file 41598_2019_56935_MOESM1_ESM.pdf]
